# Stress experienced by dental students performing clinical training in different dental disciplines: a cross-sectional study

**DOI:** 10.1093/joccuh/uiae006

**Published:** 2024-02-13

**Authors:** Rasha A Alamoush, Sereen Al-sawaeir, Dima Abu Baker, Sanaa A Aljamani, Salah A Alomoush, Mahmoud K AL-Omiri

**Affiliations:** Department of Fixed and Removable Prosthodontics, School of Dentistry, The University of Jordan, Queen Rania Street, Amman 11942, Jordan; School of Dentistry, The University of Jordan, Queen Rania Street, Amman 11942, Jordan; Department of Fixed and Removable Prosthodontics, School of Dentistry, The University of Jordan, Queen Rania Street, Amman 11942, Jordan; Restorative Department, School of Dentistry, The University of Jordan, Queen Rania Street, Amman 11942, Jordan; Department of Fixed and Removable Prosthodontics, School of Dentistry, The University of Jordan, Queen Rania Street, Amman 11942, Jordan; Department of Prosthodontics, The City of London Dental School, Canada Water, Lower Road, London, United Kingdom

**Keywords:** dental environment stress, prosthodontics clinical training, conservative clinical training, dental students

## Abstract

**Objectives:** To assess the stress level, the impact of stress factors, and discrepancies between fourth- and fifth-year undergraduate clinical students at the University of Jordan.

**Methods:** A cross-sectional study was conducted in the academic year 2022/2023. The study group included fourth- and fifth-year dental students at the University of Jordan (*n* = 382) who were asked to voluntarily fill in an online dental environment stress (DES) questionnaire. Analysis was conducted using Mann-Whitney *U* test, independent *t*-test, χ^2^ test, and Spearman ρ rank correlations. Statistical significance was inferred when *P* < .05.

**Results:** Fourth-year students reported higher levels of nervousness before treating patients for the first time. Fifth-year students faced more difficulty, higher stress, and/or fear regarding the amount of assigned work, provided treatment, completed requirements, failing the course, time to finish assignments, patient comprehensive care, and financial expenses of the course**.** Comparison between groups revealed that the fifth-year students scored higher levels of stress on the total DES score and all partial DES scores. Furthermore, the total DES questionnaire scores were significantly correlated with grade point average (GPA) among the total study sample and the prosthodontics clinical course marks among the fourth-year students’ group.

**Conclusions:** The stress level among students in clinical courses was influenced by their academic performance, gender, year level, and the type and time needed for the provided treatment. Completing course requirements was among the most commonly faced stressors. Future research endeavors might be considered to study each clinical factor, its impact on students’ stress level, and how to manage and positively improve these factors.

## Key points

Stress affects the psychological and physical health of dental students. Studies have shown that dental students are amongst the most vulnerable to stress compared with other health care professionals. Stress impacts preclinical students and progresses with their educational level.

This study aimed to assess stress levels and the discrepancy between fourth- and fifth-year dental students at the University of Jordan. Furthermore, this study investigated the impact of various general and conservative and prosthodontics course factors on students’ stress levels.

## 1. Introduction

Stress is defined as “the nonspecific response of the body to any demand”^1^ and affects psychological, emotional, and physical states as well as inducing structural changes in different parts of the brain.^2^ According to the National Center for Complementary and Integrative Health, episodic stress is considered a normal coping response.^3^ Mild stress has some beneficial effects, such as preserving homeostasis of cells/species, and facilitates memory and cognition in respect of other factors.^4^ However, long-term stress may contribute to a range of health side effects,^3^ including anxiety, depression, burnout, mental ill-health, psychosocial disturbance, physical ill-health, and gastrointestinal symptoms.^5^

Previous studies showed that dental students are among the most vulnerable to long-term stress compared with their counterparts in other medical fields.^6^^,^^7^ This might be attributed to their tendency toward perfectionism, fear of failure, exam stress, the teaching curriculum, workload, lack of leisure time, and numerous other factors. The stress factors are categorized into 5 categories: (1) living accommodation factors, (2) personal factors, (3) educational environment factors, 4) academic factors, and (5) clinical factors.^8^

The stress level increases through the progression in years of education at dental school.^9^^,^^10^ Hence, students in their clinical years are more prone to stress than those in their preclinical years.^11^ Students in their clinical years are considered dental practitioners as they start to treat patients, so they suffer from stressors that face dentists in the work environment in addition to stressors related to their academic work. The dental environment is challenging compared with other work environments and exposes practitioners to occupational health–related problems including stress, percutaneous injury, and musculoskeletal disorders.^12^ For instance, stress in the dental environment is one of the most common causes of needle-stick injuries (50%), which lead to disease transmission and harm to the practitioners.^13^

Creating a healthy and relaxed environment without hazards is a priority, as stress is one of the main health problems that dental practitioners face and it induces serious complications. Most studies in the field have focused on studying stressors regarding clinical factors in general.^14^^,^^15^ However, to the best of our knowledge, there has been no study demonstrating the specific stressors regarding different aspects of each clinic as well as the differences between clinical years regarding these factors. Consequently, this study was conducted to investigate the perceived stress levels among clinical-year dental students at the University of Jordan, focusing on prosthodontics, conservative courses, faculty administration, and general stressors. Moreover, it aimed to assess stress levels, understand the impact of various stress factors, identify differences among undergraduates across different dental clinical courses, and explore variations between fourth- and fifth-year students concerning these factors.

## 2. Methods

### 2.1. Ethical approval

The research protocol was approved by the Ethical Committee of the Faculty of Dentistry of the University of Jordan and in full accordance with the Declaration of Helsinki. All the participants were informed regarding the objectives and aims of the questionnaire, and agreed to fill in the form.

### 2.2. Study group and design

The study was a cross-sectional study conducted in the academic year 2022/2023. Participants were recruited from fourth- and fifth-year dental students in the University of Jordan. Data were collected using a self-administered online survey form. The survey was distributed during the first semester and consisted of 57 items, which have been categorized into 5 sections as follows: the first section included 13 items about sociodemographic characteristics of the participants (age, gender, scholarship, current housing, cumulative university grade point average (GPA), high school GPA, students’ grades in conservative and prosthodontics theory and preclinical courses, and if they have repeated any courses or years); the second section included 10 items regarding clinical prosthodontics factors; the third section included 9 items about clinical conservative dentistry factors; the fourth section included 14 items to assess their stress in general; and the last section included 11 items to assess students’ opinions about faculty and administrators. The participants evaluated these items based on a scale of 1-5 (1 = no stress/no fear/not difficult to 5 = extremely stressful/extremely afraid/ extremely difficult). In addition, the survey included Yes/No questions.

### 2.3. Statistical analysis

SPSS computer software (IBM SPSS Statistics v20.0; IBM Corp., USA) was used for data analysis in this study. The study variables were tested for normal distribution; then the variables were summarized accordingly. The Mann-Whitney *U* test was used to identify differences between groups and genders regarding GPA. An independent *t*-test was used to compare between groups and genders regarding dental environment stress (DES) total scores. The χ^2^ test was used to identify the significance of presence versus absence of each individual stressor among the participants in each group. Spearman ρ rank correlations were used to identify the raw correlations between individual stressors and GPA, marks obtained in clinical and theoretical prosthodontics courses, and in conservative dentistry courses. Multiple hierarchical linear regression analysis was used to examine the odds of DES. Statistical significance was assumed when *P* < .05.

A priori power analysis with a statistical power (1 – β) of 0.8, 2-tailed α probability error of .05, and an effect size of 0.3 was used to estimate the size of the study sample via G*Power (version 3.1.9.7; Heinrich-Heine University). The calculated sample size was 304 participants. Of the 519 participants invited to participate in the study, 382 responded (73.6 % response rate), and 9 participants were excluded due to incomplete responses; therefore, a total of 373 participants were included.

## 3. Results

Data from 373 participants (270 females and 103 males) were analyzed. Participants’ age ranged between 20 and 31 years (mean [SD] age = 21.8 [1.7] years). A higher percentage of fourth-year students were living with their parents, had repeated previous years, and had previously repeated courses in dentistry (*P* < .05, Table 1). In addition, fourth-year students scored a higher GPA than fifth-year students (*P* = .037, Table 1).

**Table 1:** Distribution of demographic and general variables and differences between fourth- and fifth-year participants.

		**All sample (*N* = 373)**	**Fourth year (*N* = 249)**	**Fifth year (*N* = 124)**	**Mann-Whitney *U***	***Z***	***P***
		***n* (%)**	***n* (%)**	***n* (%)**			
*Categorical nominal variables*							
Gender	Male	103 (27.6)	64 (25.7)	39 (31.5)	14 550.5	−1.168	.243
	Female	270 (72.4)	185 (74.3)	85 (68.5)			
Scholarship	Full scholarship	90 (24.1)	59 (23.7)	31 (25.0)	14 403.5	−1.112	.266
	Partial scholarship	40 (10.7)	34 (13.7)	6 (4.8)			
	National fees	156 (41.8)	102 (41.0)	54 (43.5)			
	International fees	87 (23.3)	54 (21.7)	33 (26.6)			
Current housing	With parents	254 (68.1)	183 (73.5)	71 (57.3)	12 931.5	−3.165	.002
	Without parents	119 (31.9)	66 (26.5)	53 (42.7)			
Failed academic years	No	297 (79.6)	215 (86.3)	82 (66.1)	12 317.0	−4.560	<.001
	Yes	76 (20.4)	34 (13.7)	42 (33.9)			
Failed academic courses	No	317 (85.0)	225 (90.4)	92 (74.2)	12 942.0	−4.113	<.001
	Yes	56 (15.0)	24 (9.6)	32 (25.8)			
*Continuous and scale variables*							
GPA	Median	3.1	3.2	3.1	13 396.5	−2.084	.037
	IQR	0.60	0.54	0.60			
	Range	2.0-4.0	2.1-4.0	2.0-3.97			
Clinical prostho grade	Median	7	7	7	14 570.5	−0.898	.369
	IQR	2	2	2			
	Range	2-10	2-10	3-10			
Theory prostho grade	Median	7	7	7	13 634.5	−1.861	.063
	IQR	3	3	2			
	Range	1-10	1-10	2-10			
Clinical cons grade	Median	7	7	7	14 867.5	−0.588	.557
	IQR	3	2	3			
	Range	1-10	1-10	1-10			
Theory cons grade	Median	7	7	7	15 420.0	−0.019	.985
	IQR	2	2	2			
	Range	2-10	2-10	4-10			

Cons, conservative dentistry courses; IQR, interquartile range; prostho, prosthodontics courses; Z, *z* statistic.

Table 2 presents the distribution of stressors encountered by participants and the differences between fourth- and fifth-year students in this regard. In both conservative and prosthodontics courses, fourth-year students reported higher levels of nervousness before treating patients for the first time (*P* < .05, Table 2). Meanwhile, fifth-year students faced more difficulty and higher stress levels regarding the amount of assigned work, provided treatment, completed requirements, failing the course, time to finish assignments, patient comprehensive care, and financial expenses of the course (*P* < .05, Table 2). Regarding general stressors, fifth-year students faced more difficulty and higher stress and/or fear regarding repeating a year, their ability to handle the workload, lack of follow-up from patients, availability of patients, communication with patients, competency requirements, and financial expenses of the course (*P* < .05, Table 2).

**Table 2:** Distribution of various stressors including during prosthodontics and conservative courses, and differences between fourth- and fifth-year participants (*N* = 373).

**Variables and stressors**	**Fourth year (*n* = 249)**			**Fifth year (*n* = 124)**			**Mann-Whitney *U* test**		
	**Median**	**IQR**	**Range**	**Median**	**IQR**	**Range**	**MWU**	***Z***	***P***
*Prosthodontics stressors*									
1. Stress due to amount of assigned work	3	2	1-5	4	1	1-5	10 715.5	−5.041	<.001
2. Difficulty of provided treatment	3	2	1-5	3	1	1-5	12 133.0	−3.548	<.001
3. Stress due to completing requirements	4	2	1-5	5	1	1-5	10 904.0	−4.858	<.001
4. Fear of failing the course	3	2	1-5	4	3	1-5	12 349.5	−3.228	.001
5. Stress due to lack of time to finish assignments^a^	—	—	—	—	—	—	13 957.5	−2.244	.025
6. Fear of not meeting patients’ satisfaction	3	2	1-5	3	2	1-5	15 103.5	−0.352	.725
7. Nervousness before treating a patient for the first time	3	2	1-5	3	2	1-5	13 410.0	−2.120	.034
8. Difficulty in learning the required clinical and laboratory manual skills	3	2	1-5	3	2	1-5	14 584.0	−0.904	.366
9. Stress due to comprehensive patient care	3	1	1-5	4	2	1-5	10 336.0	−5.371	<.001
10. Stress due to course financial expenses	3	2	1-5	4	2	1-5	9441.5	−6.274	<.001
*Conservative stressors*									
1. Stress due to amount of assigned work	3	2	1-5	4	2	1-5	10 919.5	−4.751	<.001
2. Difficulty of provided treatment	3	1	1-5	3	2	1-5	11 617.0	−4.053	<.001
3. Stress due to completing requirements	4	2	1-5	5	1	1-5	11 251.5	−4.449	<.001
4. Fear of failing the course	3	2	1-5	4	3	1-5	11 400.0	−4.210	<.001
5. Stress due to lack of time to finish assignments^a^	—	—	—	—	—	—	14 080.5	−2.160	.031
6. Fear of not meeting patients’ satisfaction	3	2	1-5	3	2	1-5	15 186.5	−0.266	.790
7. Nervousness before treating a patient for the first time	3	2	1-5	3	2	1-5	13 279.5	−2.257	.024
8. Difficulty in learning the required clinical and laboratory manual skills	3	1	1-5	3	1	1-5	13 863.5	−1.673	.094
9. Stress due to comprehensive patient care	3	2	1-5	4	2	1-5	10 671.5	−5.012	<.001
10. Stress due to course financial expenses^b^	—	—	—	4	2	1-5	00.0	−18.812	<.001
*General stressors*									
1. Stress due to competition with collegues	3	2	1-5	3	2	1-5	14 311.0	−1.179	.238
2. Stress due to examinations and grades	4	2	1-5	4	2	1-5	14 708.0	−0.779	.436
3. Fear of failing a year	3	3	1-5	4	2	1-5	13 065.5	−2.486	.013
4. Stress due to the workload	3	1	1-5	4	2	1-5	12 124.5	−3.494	<.001
5. Stress due to lack of follow-up from patients	4	1	1-5	4	2	1-5	11 778.5	−3.856	<.001
6. Stress due to patients not being available	4	2	1-5	5	1	1-5	11 756.0	−3.996	<.001
7. Stress due to communication with patients	3	2	1-5	4	2	1-5	11 945.5	−3.657	<.001
8. Stress due to criticism about work	3	2	1-5	3	2	1-5	15 264.5	−0.182	.855
9. Stress due to seeking patients’ confidence	3	1	1-5	3	2	1-5	15 279.5	−0.168	.867
10. Stress due to competency requirements	3	1	1-5	4	2	1-5	11 995.5	−3.632	<.001
11. Lack confidence to be a successful student	—	—	—	—	—	—	14 272.5	−1.580	.114
12. Lack confidence to be a successful dentist	—	—	—	—	—	—	14 642.0	−1.181	.238
13. Have insecurity concerning professional future	—	—	—	—	—	—	15 408.0	−0.035	.972
14. Stress due to course financial expenses	3	2	1-5	4	2	1-5	13 365.5	−2.175	.030
*Faculty and administrative stressors*									
1. Stress due to atmosphere created by faculty	5	1	1-5	5	1	1-5	12 212.5	−3.741	<.001
2. Stress due to school rules and regulation	4	2	1-5	5	1	1-5	10 629.5	−5.295	<.001
3. Expectation and reality of dental clinics	1	1	1-3	1	1	1-3	12 253.0	−3.737	<.001
4. Expectation and reality of dental school	1	1	1-3	1	1	1-3	15 022.5	−0.531	.595
5. Lack of input into the decision-making process of dental school	—	—	—	—	—	—	15 293.0	−0.181	.856
6. Stress due to lack of input into the decision-making process of dental school	3	3	1-5	3	2	1-5	13 439.0	−2.090	.037
7. Attitude toward female dental students	2	0	1-3	2	0	1-3	15 318.5	−0.147	.883
8. Presence of discrimination due to race, class status, or ethnic group	—	—	—	—	—	—	12 925.5	−3.001	.003
9. Stress due to discrimination presence	1	2	1-5	3	3	1-5	12 588.0	−3.145	.002
10. Presence of inconsistency of feedback on work between different instructors	—	—	—	—	—	—	14 249.5	−2.169	.030
11. Stress due to inconsistency of feedback on work between instructors	4	2	1-5	4	2	1-5	15 161.5	−0.291	.771

IQR, interquartile range; MWU, Mann-Whitney *U* statistic; *Z*, *z* statistic.

a The median, IQR, and range were not calculated for these questions because they are answered as yes or no.

b There are no laboratory conservative work fees for the fourth-year students.

Regarding faculty and administrative stressors, fourth-year students reported higher stress regarding expectation and reality of dental clinics and felt more inconsistency of feedback on work between different instructors (*P* < .05, Table 2). Meanwhile, fifth-year students faced more difficulty and higher stress regarding the atmosphere created by faculty, dental school rules and regulations, lacking input in the decision-making process of the school, and discrimination between students (*P* < .05, Table 2).

Comparison between groups reveals that fifth-year students scored higher levels of stress on the total DES score and all partial DES scores (*P* < .05, Table 3). In addition, females scored higher on the total DES scores as well as prosthodontics and general stressors (*P* < .05, Table 3).

**Table 3:** Differences in DES total and partial scores between groups and between genders.

**Variables**	***t*-Test for equality of means**								
	**EV**	**Between fourth- and fifth-year groups**				**Between genders**			
		***t* (*df* = 371)**	***P***	**95% CI**		***t* (*df* = 371)**	***P***	**95% CI**	
				**Lower**	**Upper**			**Lower**	**Upper**
**Prosthodontics stressors** **(10 DES items)**	1	−4.592	<.001	−4.793	−1.919	−2.605	.010	−3.586	−0.501
	2	−4.505	<.001	−4.824	−1.888	−2.682	.008	−3.546	−0.541
**Conservative stressors** **(10 DES items)**	1	−8.124	<.001	−8.086	−4.934	−1.498	.135	−3.166	0.428
	2	−7.707	<.001	−8.175	−4.845	−1.507	.133	−3.161	0.423
**General stressors** **(14 DES items)**	1	−2.527	.012	−4.429	−0.553	−3.383	.001	−5.518	−1.461
	2	−2.524	.012	−4.435	−0.547	−3.536	.001	−5.435	−1.543
**Faculty administration stressors (8 DES items)**	1	−3.430	.001	−2.256	−0.612	1.451	.148	−0.230	1.525
	2	−3.381	.001	−2.269	−0.598	1.464	.145	−0.225	1.519
**Total DES score** **(prosthodontics course)**	1	−3.899	<.001	−10.952	−3.609	−2.454	.015	−8.801	−0.970
	2	−3.829	<.001	−11.027	−3.534	−2.519	.013	−8.710	−1.061
**Total DES score** **(conservative course)**	1	−5.453	<.001	−14.198	−6.672	−2.020	.044	−8.309	−0.113
	2	−5.307	<.001	−14.309	−6.560	−2.091	.038	−8.182	−0.240

DES, dental environment stress; EV, equality of variance (1= assumed; 2 = not assumed).

Table 4 presents the significance of presence versus absence of individual stressors among participants in each group. Most of the tested individual stressors were significantly present among the study sample (*P* < .05).

**Table 4:** The significance of presence versus absence of individual stressors among the participants in each group (*N* = 373).

	**Fourth year (*n* = 249)**					**Fifth year (*n* = 124)**				
**Stressor**	**Frequency**		**χ** ^**2a**^	***df***	***P***	**Frequency**		**χ** ^**2a**^	***df***	***P***
	**Absent**	**Present**				**Absent**	**Present**			
*Prosthodontics stressors*										
1	13	236	106.000	4	<.001	3	121	56.887	4	<.001
2	15	234	108.088	4	<.001	3	121	64.548	4	<.001
3	8	241	90.257	4	<.001	1	123	115.435	4	<.001
4	40	209	5.880	4	.208	10	112	36.565	4	<.001
5	54	195	79.843	1	<.001	15	109	71.258	1	<.001
6	20	229	34.394	4	<.001	7	117	36.484	4	<.001
7	21	228	32.546	4	<.001	19	105	4.226	4	.376
8	26	223	68.008	4	<.001	11	113	29.468	4	<.001
9	15	234	57.165	4	<.001	6	118	53.984	4	<.001
10	26	223	20.177	4	<.001	5	119	61.484	4	<.001
*Conservative stressors*										
1	12	227	60.257	4	<.001	4	120	41.242	4	<.001
2	19	230	108.249	4	<.001	11	113	20.597	4	<.001
3	10	239	54.394	4	<.001	2	122	95.032	4	<.001
4	50	199	4.394	4	.355	16	108	33.742	4	<.001
5	48	201	94.012	1	<.001	13	111	77.452	1	<.001
6	24	225	61.663	4	<.001	11	113	25.597	4	<.001
7	18	231	32.867	4	<.001	22	102	5.435	4	.245
8	36	213	76.602	4	<.001	14	110	40.597	4	<.001
9	15	234	55.719	4	<.001	5	119	46.403	4	<.001
10 ^$^	—	—	—	—	—	4	120	35.919	4	<.001
*General stressors*										
1	35	214	17.406	4	.002	20	104	18.984	4	.001
2	5	244	109.976	4	<.001	5	119	48.903	4	<.001
3	31	218	13.871	4	.008	11	113	36.806	4	<.001
4	12	237	54.635	4	<.001	5	119	47.129	4	<.001
5	15	234	42.867	4	<.001	3	121	57.774	4	<.001
6	1	248	110.177	4	<.001	3	121	139.790	4	<.001
7	28	221	26.000	4	<.001	7	117	26.403	4	<.001
8	16	233	42.104	4	<.001	10	114	20.839	4	<.001
9	27	222	71.863	4	<.001	13	101	27.935	4	<.001
10	11	238	61.502	4	<.001	6	118	48.016	4	<.001
11	180	69	49.482	1	<.001	99	25	44.161	1	<.001
12	196	53	82.124	1	<.001	104	20	56.903	1	<.001
13	129	120	.325	1	.568	64	60	0.129	1	.719
14	21	228	29.775	4	<.001	10	114	31.726	4	<.001
*Faculty and administrative stressors*										
1	1	248	218.851	4	<.001	2	122	233.500	4	<.001
2	4	245	131.944	4	<.001	0	124	131.871	3	<.001
3	118	131	71.157	2	<.001	32	92	95.532	2	<.001
4	72	177	178.578	2	<.001	39	85	79.371	2	<.001
5	82	167	29.016	1	<.001	42	82	12.903	1	<.001
6	66	183	26.040	4	<.001	26	98	10.597	4	.031
7	207	42	157.036	2	<.001	97	27	38.919	2	<.001
8	159	90	19.120	1	<.001	59	65	0.290	1	.590
9	141	108	211.141	4	<.001	52	72	44.790	4	<.001
10	23	226	165.498	1	<.001	21	103	54.226	1	<.001
11	21	228	50.659	4	<.001	18	106	31.403	4	<.001

*df*, degrees of freedom.

a χ^2^ testing absence against presence of the stressor.

Statistically significant correlations were identified between the sum score of prosthodontics stressors and the GPA among the total study sample (*P* < .05, Table 5). Also, the total DES questionnaire scores were significantly correlated with GPA among the total study sample (*P* < .05, Table 5). However, no correlations were identified between DES total/prosthodontics stressors scores and the GPA, prosthodontics clinical course marks, and prosthodontics theory course marks among the fifth-year students’ group (*P* > .05, Table 5).

**Table 5:** Correlations of total, partial, and individual dental environment stress scores with GPA and clinical and theory course grades during the prosthodontics and conservative dentistry courses among the study sample.

**Stressor**	**Sp rho**	**Total study sample (*N* = 373)**			**Fourth year (*n* = 249)**			**Fifth year (*n* = 124)**		
		**GPA**	**Clinical** ^**a**^	**Theory** ^**b**^	**GPA**	**Clinical** ^**a**^	**Theory** ^**b**^	**GPA**	**Clinical** ^**a**^	**Theory** ^**b**^
*Prosthodontics stressors*										
1	*R*	−0.135	−0.078	−0.032	−0.188	−0.145	−0.105	0.079	0.026	0.030
	*P*	.009	.133	.543	.003	.022	.097	.382	.776	.739
2	*R*	−0.145	−0.088	−0.056	−0.202	−0.145	−0.139	0.012	-0.018	0.051
	*P*	.005	.089	.283	.001	.023	.028	.893	.845	.574
3	*R*	−0.148	−0.134	−0.043	−0.145	−0.171	−0.079	−0.086	−0.109	−0.050
	*P*	.004	.010	.409	.022	.007	.213	.344	.228	.581
4	*R*	−0.282	−0.151	−0.190	−0.331	−0.206	−0.260	−0.137	−0.057	−0.105
	*P*	.000	.003	.000	.000	.001	.000	.130	.531	.247
5	*R*	0.032	−0.005	0.064	0.040	−0.070	0.032	0.057	0.141	0.103
	*P*	.533	.923	.221	.533	.273	.612	.529	.119	.257
6	*R*	−0.008	−0.021	0.032	−0.035	−0.082	−0.010	0.023	0.117	0.130
	*P*	.884	.679	.537	.580	.197	.874	.797	.197	.150
7	*R*	−0.057	−0.057	−0.045	−0.106	−0.086	−0.060	−0.005	0.007	0.019
	*P*	.276	.273	.389	.096	.175	.343	.953	.941	.835
8	*R*	−0.129	−0.148	−0.060	−0.185	−0.229	−0.121	−0.015	0.004	0.042
	*P*	.012	.004	.251	.003	.000	.057	.872	.966	.647
9	*R*	−0.087	−0.008	0.012	−0.055	−0.010	−0.005	−0.078	−0.025	−0.030
	*P*	.094	.884	.813	.388	.875	.935	.388	.784	.745
10	*R*	−0.042	0.130	0.069	−0.086	0.087	−0.021	0.110	0.201	0.139
	*P*	.422	.012	.181	.175	.172	.738	.223	.026	.122
Sum score for the 10 prosthodontics stressors	*R*	−0.170	−0.087	−0.047	−0.218	−0.160	−0.125	−0.025	0.036	0.029
	*P*	.001	.094	.364	.001	.012	.048	.786	.694	.746
Total score for the DES	*R*	−0.121	−0.064	−0.015	−0.134	−0.125	−0.048	−0.065	0.020	−0.025
	*P*	.019	.218	.777	.035	.048	.447	.473	.829	.784
*Conservative stressors*										
1	*R*	−0.077	−0.140	0.011	−0.046	−0.111	0.003	−0.067	−0.183	0.023
	*P*	.136	.007	.831	.468	.080	.968	.461	.041	.797
2	*R*	−0.176	−0.136	−0.100	−0.176	−0.068	−0.126	−0.132	−0.225	−0.067
	*P*	.001	.009	.054	.005	.282	.048	.144	.012	.461
3	*R*	−0.070	−0.065	−0.027	−0.019	−0.026	−0.038	−0.102	−0.128	−0.016
	*P*	.180	.207	.602	.760	.685	.546	.258	.156	.864
4	*R*	−0.183	−0.094	−0.164	−0.178	−0.010	−0.205	−0.124	−0.205	−0.099
	*P*	.000	.071	.001	.005	.872	.001	.170	.022	.276
5	*R*	−0.006	−0.070	0.026	0.025	−0.059	0.037	−0.037	−0.091	−0.003
	*P*	.907	.180	.619	.693	.352	.558	.685	.317	.977
6	*R*	−0.076	−0.095	−0.060	−0.089	−0.079	−0.090	−0.071	−0.124	0.002
	*P*	.141	.066	.248	.160	.213	.155	.433	.172	.980
7	*R*	−0.069	−0.112	−0.023	−0.087	−0.054	−0.070	−0.080	−0.214	0.075
	*P*	.182	.030	.653	.170	.393	.272	.374	.017	.410
8	*R*	−0.141	−0.182	−0.084	−0.161	−0.209	−0.112	−0.080	−0.126	−0.024
	*P*	.006	.000	.105	.011	.001	.079	.376	.164	.791
9	*R*	−0.105	−0.059	−0.029	−0.072	0.004	−0.053	−0.103	−0.160	0.030
	*P*	.043	.255	.575	.260	.955	.402	.257	.075	.738
10^c^	*R*	−0.103	−0.036	0.007	-.	-	-	0.002	−0.046	0.069
	*P*	.048	.491	.888	-	-	-	.979	.609	.448
Sum score for the 10 conservative stressors	*R*	−0.156	−0.136	−0.068	−0.124	−0.081	−0.107	−0.136	−0.241	−0.012
	*P*	.002	.009	.189	.051	.203	.093	.133	.007	.894
Total score for the DES	*R*	−0.118	−0.066	−0.056	−0.091	0.000	−0.083	−0.116	−0.173	−0.022
	*P*	.023	.205	.278	.151	.995	.190	.199	.054	.804

DES, dental environment stress; GPA, grade point average; *R*, Spearman correlation coefficient; Sp rho, Spearman rho correlation test.

a Clinical = clinical course mark.

b Theory = theory course mark.

c There are no laboratory conservative work fees for the fourth-year students.

Moreover, statistically significant correlations were identified between the sum score of conservative stressors and the GPA (*P* = .002) and the conservative clinical course marks (*P* = .009), among the total study sample as well as the conservative clinical course marks (*P* = .007, Table 5). However, no correlations were identified between the conservative stressor scores and the GPA, conservative clinical course marks, and conservative theory course marks among the fourth-year students (*P* > .05, Table 6). In addition, the total DES questionnaire scores had significant correlation with the GPA of the total study sample (P = 0.023, Table 5).

**Table 6:** Hierarchical regression analysis to predict dental environment stress and GPA among the study sample (*N* = 373).

**Dependent variable**	**Predictors** ^**a**^	**Unstand Co**		**Stand Co**	***t***	***P*** ^**b**^	**95% CI for β**	
		**β**	**SE**	**β**			**Lower bound**	**Upper bound**
*Regression for GPA considering stress during prosthodontics course*								
GPA *R*^2^ = 0.135	(Constant)	4.945	0.288	—	17.157	<.001	4.378	5.512
	Group^c^	.039	0.045	.046	0.860	.390	−.050	.128
	Age	−.063	0.012	−.272	−5.098	.000	−.087	−.039
	Gender	.013	0.045	.014	0.288	.773	−.075	.101
	Scholarship	.005	0.018	.013	0.275	.783	−.031	.041
	Current housing	−.152	0.044	−.175	−3.460	.001	−.238	−.066
	DES during prosthodontics course	−.003	0.001	−.124	−2.465	.014	−.005	−.001
*Regression for GPA considering stress during conservative course*								
GPA *R*^2^ = 0.132	(Constant)	4.895	0.285	—	17.162	<.001	4.334	5.456
	Group^c^	.044	0.046	.051	0.943	.346	−.047	.135
	Age	−.063	0.012	−.271	−5.077	.000	−.087	−.039
	Gender	.010	0.045	.011	0.222	.824	−.078	.098
	Scholarship	.005	0.018	.014	0.284	.777	−.031	.041
	Current housing	−.150	0.044	−.174	−3.422	.001	−.237	−.064
	DES during conservative course	−.003	0.001	−.113	−2.196	.029	−.005	.000
*Regression for dental environment stress during prosthodontics course*								
DES *R*^2^ = 0.080	(Constant)	106.241	16.168	—	6.571	<.001	74.446	138.035
	Age	−.669	0.564	−.067	−1.185	.237	−1.779	.441
	Gender	5.180	1.966	.134	2.635	.009	1.313	9.046
	Scholarship	1.210	0.802	.076	1.509	.132	−.367	2.787
	Current housing	−1.061	1.968	−.029	−0.539	.590	−4.930	2.809
	Group^c^	7.973	1.966	.217	4.056	<.001	4.107	11.838
	GPA	−5.644	2.289	−.132	−2.465	.014	−10.146	−1.142
*Regression for dental environment stress during conservative course*								
DES *R*^2^ = 0.109	(Constant)	96.260	16.618	—	5.793	<.001	63.582	128.938
	Age	−.625	0.580	−.060	−1.078	.282	−1.766	.515
	Gender	4.775	2.021	.118	2.363	.019	.801	8.748
	Scholarship	1.456	0.824	.088	1.767	.078	−.164	3.077
	Current housing	−.467	2.023	−.012	−0.231	.817	−4.445	3.510
	Group^c^	10.966	2.020	.286	5.428	<.001	6.993	14.939
	GPA	−5.168	2.353	−.116	−2.196	.029	−9.795	−.541

DES, dental environment stress; GPA, grade point average; *R*^2^, coefficient of determination; Stand Co, standardized coefficient; Unstand Co, unstandardized coefficient; β, beta statistic.

a Final models are presented with group, age, gender, scholarship, and current housing, which were included in the first block of the regression model, whereas the DES and GPA scores were included in the second block.

b Group = fourth-year student and fifth-year student groups.

c Two-tailed probability value.

The hierarchical multiple linear regression analysis of the GPA revealed that higher odds of inferior academic performance were associated with higher stress during both prosthodontics and conservative dentistry courses (*P* < .05, Table 6). Also, a lower GPA was associated with older age of participants and living without parents (*P* < .05, Table 6). Furthermore, higher odds of stress were associated with being a female, being a fifth-year student, and having lower academic performance (*P* < .05, Table 6).

## 4. Discussion

Many studies have assessed stress levels amongst dental students and their impact on mental, psychological, and physical health using different methods (eg, DES, general health questionnaire) and scales such as perceived stress scale (PSS) and visual analog scale (VAS), whereas others have studied biological stress markers including cortisol and immunoglobulin A levels.^16^^-^^19^

The undergraduate dental degree at the University of Jordan is a 5-year course, the first 2 years of which comprise medical sciences and 3 basic dental subjects. The third year covers medical sciences and dental subjects, mainly conservative dentistry and prosthodontics, including preclinical training. In their fourth and fifth years, students start to treat patients. This study aimed to assess the perceived stress among dental students in their clinical years at the University of Jordan regarding prosthodontics and conservative courses, as well as faculty administration and general stressors.

### 4.1. Gender

The present study has shown that there are gender variations, as female students scored higher level of stress than males in the total DES scores as well as in prosthodontics clinical training and general stressors. Previous studies showed that stress levels in female dental students are higher than those in male students,^9^^,^^14^ coinciding with the fact that stress level is higher in females in the general population due to the hormonal, physiological, and stressor variations between the genders.^20^

### 4.2. GPA

There was a significant negative relationship between GPA and DES: the higher the student’s GPA the lower their level of stress and vice versa, in line with other studies.^6^^,^^21^ A possible explanation is that students with high GPAs are usually well prepared for exams and have better time management skills. However, stress may affect students’ lifestyle, and induce anxiety, exhaustion, and depression or reduced accomplishment,^5^^,^^8^ which might impact their study capacity and academic motivation so their GPAs will be reduced.^22^

Regarding prosthodontics clinical training, a significant negative relationship was found between GPA and stress; this was attributed to several stressors including amount of assigned work, difficulty of provided treatment, and fear of failing the course. It is noteworthy that fear of failing the course was the prosthodontics stressor most negatively correlated with the GPA, and fear of failing was mentioned in other studies to be one of the most important clinical stressors.^6^^,^^18^^,^^23^ On the other hand, in conservative clinical training, the negative relationship was attributed to the following stressors: difficulty of provided treatment, fear of failing the course, difficulty in learning the required clinical and laboratory manual skills, stress due to comprehensive patient care, and stress due to course financial expenses. Also, fear of failing was the conservative stressor most negatively correlated with the GPA.

### 4.3. Prosthodontics clinical training stressors

The most stressful factor was completing requirements in both years, in line with results of previous studies that reported that completing the requirements of the clinics is the most stressful factor during the clinical years.^8^^,^^9^ Although fifth-year students reported higher stress in respect of this factor than fourth-year students, this significant difference between the years could be explained by the higher number of prosthodontics clinical requirements in the fifth year. In a previous study,^24^ fear of failing courses in general was one of the important stressors among dental students, regardless of their year of study or the specific course.

In the current study, it is surprising that the fear of failing prosthodontics courses was not significantly present as a stressor among fourth-year students, unlike fifth-year students who significantly considered failing prosthodontics courses as a stressor, which could be explained by the larger number of requirements compared with those of the fourth year. In addition, fourth-year students are distributed as partners, and it was shown in a published study that clinical pairing was beneficial in conservative and prosthodontics clinical training and contributed to reducing stress and strain levels.^25^ Meanwhile, the stress regarding treating patients for the first time was not significantly present among fifth-year students, who were familiar with treating new patients after a year of practicing, unlike the fourth-year students, who scored a significant stress level regarding this factor. This result was in agreement with a previous study that showed that the patient training factor of the DES scored higher among fourth-year students than fifth-year students.^26^

### 4.4. Conservative clinical training stressors

Fifth-year students experienced higher stress levels compared with fourth-year students regarding conservative clinical training. This could be attributed to several factors, including increased workload, higher laboratory expenses, and the challenges associated with certain tasks, such as performing root canal treatments on molars, which has been acknowledged as complex and difficult for undergraduate students to do.^27^ The most stressful factor related to conservative clinical training in both years was completing the requirements. It is noteworthy that the amount of assigned work and course financial expenses were recorded as the second most present stressors among fifth-year students, who are required to provide fixed prostheses treatments that need financial coverage for the laboratory cost. Students’ fear of noncompletion of the requirements and the huge amount of assigned work may subject them to pressure, causing them to compromise their principles and ethics in their work.^28^ As per prosthodontics clinical courses, the fear of failing conservative clinical courses was not significantly present as a stressor among fourth-year students, just as the stress regarding treating patients for the first time was not significantly present among fifth-year students.

In this study, it was noted that stressors in the prosthodontics clinical course were higher than those in the conservative clinical course. This discrepancy might be attributed to several factors, including the longer duration of prosthodontics procedures, the involvement of older patients, and the responsibility of students to identify edentulous patients for complete denture or caries-free patients for removable partial denture treatments, or to prepare them accordingly. The success of treatment in prosthodontics is multifactorial and is closely affected by patient satisfaction and cooperation.^29^ Additionally, prosthodontics involves intricate procedures and extensive knowledge, and a published study has shown that tasks like border molding, jaw relations, and final impressions are among the most stressful procedures for dental students in the prosthodontics clinic.^30^

### 4.5. General stressors

The most stressful general factor in both year groups was the inability of the patients to attend at the appointment time for treatment, in line with a similar study.^31^ On the other hand, examinations and grades were reported to be the cause of most stress among dental students despite their years of study.^32^^,^^33^ It is noteworthy that a previous study of dental students at the University of Jordan in 2011, which was updated in 2016, coincided with our findings that the stressor relating to patients being late or not showing up for their appointment gained a higher level than that for examinations and grades.^9^^,^^10^ This requires the college to improve attempts to organize the process of obtaining patients, and to provide the means to encourage them to turn up for their appointments and subsequently reduce stress levels among students. Also, patient lack of awareness about dental procedures may be the reason for their delay or lack of commitment, as a previous study demonstrated that there was no significant difference in stress levels between patients who attended students’ dental clinics and specialists’ clinics.^34^ Further, it assessed the difference between pre- and posttreatment stress among new patients who sought treatment in students’ dental clinics and found that pretreatment stress levels were higher than posttreatment stress, even regarding examination and taking x-rays, possibly because the procedures were not as bad as the patients had anticipated. A study conducted in the 2021/2022 academic year at the University of Jordan showed that patients who attended dental students’ clinics were satisfied with the provided treatment.^35^

Fifth-year students also considered lack of patient follow-up as the highest stressor because fifth-year requirements need more time to be accomplished, such as endodontic treatment of molars as well as crown and bridge preparation. Also, they have to present 1 comprehensive case at the end of fifth year, so patients’ factors played an important role. Despite the benefits of the comprehensive-based teaching mentioned in a study conducted at the University of Jordan, it caused apprehension among students due to patient commitment–related factors, and has also contributed to increased stress among students.^36^

Neither fourth-year students nor fifth-year students had insecurities about their professional future or lacked the confidence to be successful students or dentists, with no significant difference between the years. These factors were categorized as self-efficacy factors in previous studies, which found a clear stressful effect on the students.^26^^,^^33^ This was contrary to our study, which showed a positive indication of students’ confidence in the educational level they had reached. However, it could also be attributed to lack of exposure to ‘real’ life and the work environment, which may diminish their concerns about the future. That is why the faculty started to involve the students in an outreach system during the second semester of the 2022/2023 academic year.

### 4.6. Faculty administration stressors

This factor scored the lowest in comparison with other stressor factors, in line with a study that showed that the faculty administration factor was the lowest stress factor.^33^ However, another study showed that faculty administration had a high score.^31^ This discrepancy could be attributed to the differences between staff, rules, and other factors among the universities.

The atmosphere created by the faculty was the highest faculty-related stressor among fourth-year dental students, in line with the result of a previous study.^37^ However, fifth-year students faced more stress regarding this factor than fourth-year students, which requires the college to take appropriate action, make efforts to reduce the pressure on them, and improve communication between faculty members and students. Regarding this, previous studies clarified certain points indicating that the student-faculty relationship has a positive effect on students’ performance,^38^ that faculty’s support reduces stress levels,^39^ and that improving communication between dental students and instructors may reduce stress levels.^40^ The most recorded faculty-related stressor among fifth-year students was school rules and regulations. Similarly, this factor was considered one of the major stressors among Indian students.^15^

What is striking in the findings was that the faculty attitude toward females was significantly absent as a stressor among fourth- and fifth-year students. Furthermore, no notable instances of discrimination due to race, class status, or ethnic group were reported among fourth-year students. It is worth mentioning that a positive educational environment should be devoid of gender and racial discrimination,^28^ so the number of students who perceived such discriminations should not be ignored, even if they are a minority.

### 4.7. Conclusions

Within the limitations of this study, the following can be concluded. The stress level among students in clinical courses was influenced by their academic performance, gender, year level, and the type and time needed for the provided treatment. Completing course requirements was among the most common stressors. Hence, and in light of our findings, future research endeavors might be considered to study each clinical factor, its impact on students’ stress levels, and how to manage and positively improve these factors.

## Author contributions

R.A.A.: conceptualization, writing; S.A.-s.: data collection, writing; D.A.B: writing, final revision; S.A. Aljamani: data collection; S.A. Alomoush: final revision; M.K.A.-O.: data analysis.

## Funding

No funding was acquired for this study.

## Conflicts of interest

The authors declare no conflicts of interest.

## Data availability

Data included in this study are available upon request.
