# Correction to: Stress experienced by dental students performing clinical training in different dental disciplines: a cross-sectional study

**DOI:** 10.1093/joccuh/uiae022

**Published:** 2024-05-10

This is a correction to: Rasha A Alamoush, Sereen Al-sawaeir, Dima Abu Baker, Sanaa A Aljamani, Salah A Alomoush, Mahmoud K AL-Omiri, Stress experienced by dental students performing clinical training in different dental disciplines: a cross-sectional study, Journal of Occupational Health, Volume 66, Issue 1, January-December 2024, uiae006, https://doi.org/10.1093/joccuh/uiae006

In the originally published version of this article, one of author Mahmoud K. AL-Omiri’s affiliations was inadvertently omitted.

The author’s affiliations have been corrected to read as follows:

^1^Department of Fixed and Removable Prosthodontics, School of Dentistry, The University of Jordan, Queen Rania Street, Amman 11942, Jordan,

and

^4^Department of Prosthodontics, The City of London Dental School, Canada Water, Lower Road, London, United Kingdom.

